# Spermidine Is Critical for Growth, Development, Environmental Adaptation, and Virulence in *Fusarium graminearum*

**DOI:** 10.3389/fmicb.2021.765398

**Published:** 2021-11-19

**Authors:** Guangfei Tang, Haoxue Xia, Jingting Liang, Zhonghua Ma, Wende Liu

**Affiliations:** ^1^State Key Laboratory for Biology of Plant Diseases and Insect Pests, Institute of Plant Protection, Chinese Academy of Agricultural Sciences, Beijing, China; ^2^State Key Laboratory of Rice Biology, Key Laboratory of Molecular Biology of Crop Pathogens and Insects, Institute of Biotechnology, Zhejiang University, Hangzhou, China

**Keywords:** *Fusarium graminearum*, polyamine biosynthesis, spermidine, DON (deoxynivalenol), virulence

## Abstract

Putrescine, spermidine, and spermine are the most common natural polyamines. Polyamines are ubiquitous organic cations of low molecular weight and have been well characterized for the cell function and development processes of organisms. However, the physiological functions of polyamines remain largely obscure in plant pathogenic fungi. *Fusarium graminearum* causes Fusarium head blight (FHB) and leads to devastating yield losses and quality reduction by producing various kinds of mycotoxins. Herein, we genetically analyzed the gene function of the polyamine biosynthesis pathway and evaluated the role of the endogenous polyamines in the growth, development, and virulence of *F. graminearum*. Our results found that deletion of spermidine biosynthesis gene *FgSPE3* caused serious growth defects, reduced asexual and sexual reproduction, and increased sensitivity to various stresses. More importantly, Δ*Fgspe3* exhibited significantly decreased mycotoxin deoxynivalenol (DON) production and weak virulence in host plants. Additionally, the growth and virulence defects of Δ*Fgspe3* could be rescued by exogenous application of 5 mM spermidine. Furthermore, RNA-seq displayed that FgSpe3 participated in many essential biological pathways including DNA, RNA, and ribosome synthetic process. To our knowledge, these results indicate that spermidine is essential for growth, development, DON production, and virulence in *Fusarium* species, which provides a potential target to control FHB.

## Introduction

Polyamines (PAs) are ubiquitous polycations found in both eukaryotic and prokaryotic organisms. The most common polyamines widely found in all organisms are putrescine (Put), spermidine (Spd), and spermine (Spm) (Igarashi and Kashiwagi, 2019). The Put is produced by ornithine decarboxylase 1 (*SPE1*) from the amino-acid L-ornithine. The synthesis of Spd and Spm is mainly catalyzed by spermidine synthase (*SPE3*) and spermine synthase (*SPE4*), respectively. Both reactions require the previous decarboxylation of S-adenosylmethionine (SAM) which is catalyzed by S-adenosylmethionine decarboxylase (*SPE2*). SAM donates the aminopropyl group for the conversion of Put into Spd, and subsequently of Spd into Spm (Vanrell et al., 2013).

Polyamine biosynthesis (Spe) genes have been cloned and sequenced in *Saccharomyces cerevisiae*. The *SPE1*, *SPE2*, and *SPE3* genes are important for growth in a polyamine-free medium (Cohn et al., 1980; Balasundaram et al., 1991; Hamasaki-Katagiri et al., 1997). The *SPE4* gene was also identified, and it was found that Spm played a minor role in growth in *S. cerevisiae* (Hamasaki-Katagiri et al., 1998). However, ornithine decarboxylase 1 was lost in the model plant *Arabidopsis thaliana* (Hanfrey et al., 2001). Therefore, plants take an alternative pathway to produce Put. Arginine serves as a precursor, first forming agmatine by arginine decarboxylase (ADC), and then converts to N-carbamoylputrescine through agmatine iminohydrolase. Subsequently, N-carbamoylputrescine amidohydrolase catalyzes and generates Put (Hanfrey et al., 2001). In *A. thaliana*, two genes, *ADC1* and *ADC2*, have been identified as regulating ADC synthesis. *AdoMetDC1* and *AdoMetDC2* are recognized to catalyze SAM production. Spd is synthesized by two genes (*SPDS1* and *SPDS2*), *ACL5* and *SPMS* are essential for Spm synthesis (Hanzawa et al., 2000, 2002; Hummel et al., 2004). *ADC1* and *ADC2* have redundant functions in the responses to plant stress in *Arabidopsis*. The *adc1* and *adc2* double mutants lead to reduce Put accumulation and then reduce freezing tolerance (Lou et al., 2020). The spermidine synthase genes play an important role in embryonic development in *A. thaliana* (Imai et al., 2004b). Consistent with *S. cerevisiae*, the gene encoding spermine synthase is also not essential for the survival of *A. thaliana* (Imai et al., 2004a). In general, the role of polyamine in *S. cerevisiae* and *A. thaliana* has not been identified and characterized to any significant extent. Other organisms such as bacteria and actinobacteria may produce polyamines as well (Bashan et al., 2004; Kumar et al., 2019). The polyamines-produced organisms have important roles in increasing yield, and biomass and reducing abiotic stress effects (El-Tarabily et al., 2020, 2021; Mathew et al., 2020). In filamentous fungi *Aspergillus flavus*, the results showed that inactivation of *SPE3* clearly reduced conidiation, aflatoxin production, and virulence on maize seed (Majumdar et al., 2018). Recently, Rocha et al. (2020) illustrated that Spm was essential for development and pathogenicity during the early stages of infection in *Magnaporthe oryzae*. However, the functions of the genes in the polyamine biosynthesis pathway in *Fusarium graminearum* have not been demonstrated. Therefore, further elucidation of the mechanisms underlying polyamine biosynthesis is imperative for the development of novel strategies to manage *F. graminearum*-caused diseases.

Polyamines are required for many fundamental processes of nucleic acid synthesis, the stabilization of RNA, DNA, and protein structure (Igarashi and Kashiwagi, 2000). Put and Spd are predominant polyamines that are often found in fungi, while Spm is absent from some fungal genera (Valdés-Santiago et al., 2012). Polyamine depletion causes a serious growth defect in some organisms, whereas excess polyamines in cells show cytotoxicity, indicating an exact regulation of polyamine metabolism is a necessity for cellular processes (Casero et al., 2018). Additionally, polyamines have been identified to regulate various biological processes, including vegetative growth, spore germination, and stress tolerance and virulence of plant and animal fungal pathogens (Ruiz-Herrera, 1994; Khurana et al., 1996).

Fusarium head blight (FHB), also known as head scab disease is caused by *F. graminearum*, and is a destructive fungal disease of cereal grains worldwide (Dean et al., 2012; Chen et al., 2019). In addition to substantial yield losses, mycotoxin contamination, such as deoxynivalenol (DON), nivalenol, and zearalenone (ZEN), exerts an adverse threat to the health of humans and animals (Chen et al., 2019). DON (also known as vomitoxin) is one of the most commonly occurring trichothecenes in cereals and cereal-derived food products. This can result in feed refusal, immune dysregulation, teratogenicity, and reproductive defects in mammals (Pestka, 2010; Lee and Ryu, 2017). Meanwhile, DON, as a vital virulence factor, plays a vital role in the pathogenic process. It is very important to explore the mechanism of DON synthesis for the control of FHB. DON production was affected by several environmental factors including carbon, nitrogen, pH, and light (Chen et al., 2019). Recently, using a large-scale nutrient profiling, Gardiner et al. (2009a, 2010) revealed that exogenous Put can significantly increase DON production and further testified that Put produced by plants might play a role in inducing DON biosynthesis during the *F. graminearum* infection process. However, the molecular mechanisms in fungi linking the endogenesis polyamine biosynthetic pathway to DON production and pathogenicity are not clear in *F. graminearum*. In this study, four genes (*FgSPE1*, *FgSPE2*, *FgSPE3*, and *FgSPE4*) which were involved in the polyamine biosynthetic pathway, were identified and genetically analyzed in *F. graminearum*. Further, our study evaluated the function of polyamines, and demonstrated that spermidine was important for hyphal growth, conidial production, DON biosynthesis, environmental stress response, and virulence in this important phytopathogenic fungus.

## Materials and Methods

### Fungal Strains and Growth Assays

The wild-type strain PH-1 (NRRL 31084) was chosen as parental strain for generating all deletion mutants (Cuomo et al., 2007). All strains were cultured on complete medium (CM), potato dextrose agar (PDA), minimal medium (MM), and yeast extract peptone dextrose (YEPD) medium at 25°C to assay growth rate.

### Generation of Gene Deletion and Complementation Strains

The gene deletion mutants were constructed by double-joint PCR method, the primer pairs which were used to amplify the upstream and downstream sequence of the targeted gene are shown in Supplementary Table 1. The resulting products were then transformed into the protoplasts of wild-type PH-1 as described previously (Tang et al., 2020). To complement the Δ*Fgspe3*, the Fgspe3-GFP cassette (GFP, green fluorescent protein) was constructed, a Fgspe3 fragment which included the promoter and gene fragment (without stop codon) was amplified from wild-type genomic DNA with specific primers listed in Supplementary Table 1. The resulting complementation fragments and Xho1 digested pYF11 vector were co-transformed into yeast XK1-25. The recombined FgSPE3-GFP fusion vector was transformed into *Escherichia coli* for amplification and then transformed into the Δ*Fgspe3* mutant, and the generating transformant was named as the Δ*Fgspe3*-C. By means of the same methods, the FgSPE1-GFP, FgSPE2-GFP, and FgSPE4-GFP recombinant plasmids were transformed into the mutants, respectively, and generated the Δ*Fgspe1*-C, Δ*Fgspe2*-C, and Δ*Fgspe4*-C complementation strains.

### Conidiation, Perithecium Formation Assays

For conidia production, fresh mycelial plugs of each tested strain were inoculated in liquid carboxymethyl cellulose (CMC) medium (0.1% CMC, 2 mM MgSO_4_, 4 mM NH_4_NO_3_, 10 mM KH_2_PO_4_, and 1 L water). The 50-mL flasks were cultured in a shaker (180 rpm) for 4 days at 25°C. The number of conidia for each strain in the medium was counted using a hemacytometer (Sigma-Aldrich, United States). The conidial morphology was observed by Calcofluor White (CFW) staining and photographed with a confocal microscope (Gottingen, Niedersachsen, Germany). Perithecium production was inoculated on carrot agar medium (CA) with a 12 h/12 h light/dark cycle at 25°C.

### Stress Sensitivity Tests

To determine the growth rate under different stress conditions for deletion mutants, fresh mycelia plugs (5 mm in diameter) of each strain were inoculated on PDA medium containing various stress compounds described in the figure legends. Exogenous NO_3_^–^, NH_4_^+^, Put, Spd, and Spm (Sigma-Aldrich, United States) at 5 mM were separately added into minimum medium minus nitrogen (MM-N) medium to evaluate the growth of the Δ*Fgspe* mutants. MM-N medium which did not include any nitrogen source served as a control.

### Pathogenicity and Deoxynivalenol Production Assay

Conidia were collected from a CMC medium and adjusted to 10^5^ conidia/mL with sterile water. A 10-μL conidial suspension was inoculated into a spikelet of a 6-week-old flowering wheat head. For each strain, 15 replicates were injected into spikelets for analysis. The infected spikelet was characterized 15 days after inoculation. To further analyze the pathogenicity of the Δ*Fgspe* mutants in detail, a fresh mycelia plug of wild-type and mutants was placed on fresh corn silks. Then in 5 days after incubation at 25°C, the extent of discoloration was recorded. For wheat leaf infection assays, the 4-week-old fresh leaves were inoculated with mycelial plugs at 25°C. Pictures were taken 6 days after inoculation. For wheat coleoptile infection assays, 3-day-old seedlings of wheat were used. Conidial suspension was injected and recorded symptoms 7 days after inoculation. For each strain, there were 15 replicates in each experiment.

For detection of DON production, Δ*Fgspe3* strain was grown in liquid trichothecene biosynthesis induction (TBI) medium at 28°C for 1 week in the dark. The supernatant was harvested by three layers of filter paper and then quantified by a liquid chromatography tandem–mass spectrometry (LC-MS/MS) system (Tang et al., 2018).

### Quantitative Real-Time PCR

The wild-type and Δ*Fgspe3* were cultured into TBI medium for 2 days at 28°C. The mycelia of each strain were harvested, and then total RNA was isolated using the TaKaRa RNAiso Extraction Kit, and then used RevertAid H Minus First Strand cDNA Synthesis Kit to synthesize cDNA (Fermentas Life Sciences, Burlington, ON, Canada). The transcriptional levels of *TRI1*, *TRI5*, and *TRI6* of PH-1 were determined by quantitative reverse transcription-polymerase chain reaction (RT-qPCR) using the method previously described (Liu et al., 2019). *ACTIN* (*FGSG_07335*) gene was served as a control. The primers were listed in Supplementary Table 1.

### Confocal Microscopy Observation

The conidia of deletion mutant were stained by 0.1 mg/mL CFW for 30 s and observed with a fluorescent microscope (Gottingen, Niedersachsen, Germany). Hyphal branching patterns were examined with a Nikon microscope.

### RNA-Seq Analysis

For deep transcriptome sequencing, 2-day-old mycelia of wild-type and Δ*Fgspe3* strain were harvested from liquid YEPD shaken at 180 rpm, washed three times, and ground in liquid nitrogen. For each strain, there are three biological replicates independently. Total RNA was isolated, and cDNA libraries were constructed by NEBNext^®^ Ultra^TM^ Directional RNA Library Prep Kit (New England Biolabs, Ipswich, MA, United States) using the manufacturer’s instructions. The cDNA libraries were sequenced by an Illumina Hiseq Novaseq platform with paired-end reads (150 bp) by Novogene Corporation (Beijing, China). Reads were mapped to the genome of *F. graminearum* PH-1 strain. DESeq R package was used for differential expression gene analysis by Novogene Corporation (Beijing, China). Genes with log_2_ Fold change > 1 or < −1 and *P*-value < 0.05 were divided into differentially expressed. Raw data of the RNA-seq experiments generated in this article were deposited in the National Center for Biotechnology Information under the accession number PRJNA759842^1^.

### Statistical Analysis

Graphs were plotted by GraphPad Prism 7.0 (GraphPad Software, San Diego, CA, United States). The statistical experiments were evaluated using SPSS Statistics 21.0 software. Statistical analysis was performed one-way ANOVA and Fisher’s LSD test (*P* < 0.05).

## Results

### Identification of Polyamine Biosynthesis Pathway Genes in *Fusarium graminearum*

**FIGURE 1:**
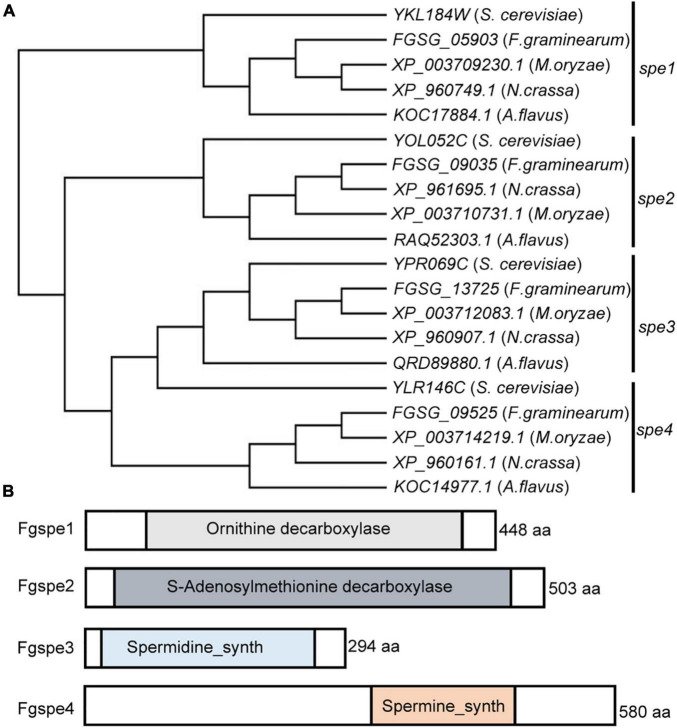
Identification of the Fgspe proteins in *F. graminearum*. **(A)** Phylogenetic analysis of SPEs from *F. graminearum*, *S. cerevisiae*, and other filamentous fungi. Protein sequences of Fgspe1–4 paralogues were aligned by CLUSTALW, and a neighbor-joining tree was constructed by MEGA 7.0. The names of all proteins are shown in the figure. **(B)** Schematic drawing of Fgspe superfamily proteins members in *F. graminearum*. Conserved domains are indicated in the figure.

Combined with the other fungi, plants, and animals, we firstly inferred the pathway of polyamine biosynthesis in *F. graminearum* (Supplementary Figure 1). Further, using the *S. cerevisiae* polyamine biosynthesis (Spe) protein sequence to query *F. graminearum* genome, the heterologous genes involved in polyamine biosynthesis which included *FGSG_05903* (designated as Fgspe1), *FGSG_09035* (designated as Fgspe2), *FGSG_13725* (designated as Fgspe3), and *FGSG_09525* (designated as Fgspe4) were revealed in this fungus. To further confirm the Spe protein evolution relationships within the organism, phylogenetic analysis of the Spe protein sequence was performed and indicated that the Spes protein was highly conserved in fungi. Interestingly, most Spe homologs were notably clustered into one distinct subclade (Figure 1A). Meanwhile, the InterPro database and tools for protein domain analysis indicated that FgSpes protein harbored conserved domains that are unique to Spe proteins (Figure 1B). In general, The domain characteristic and the phylogenetic tree indicated Spe homologs proteins were conserved and might have similar biological functions in other fungi.

### Spermidine Is Important for Hyphal Growth

**FIGURE 2:**
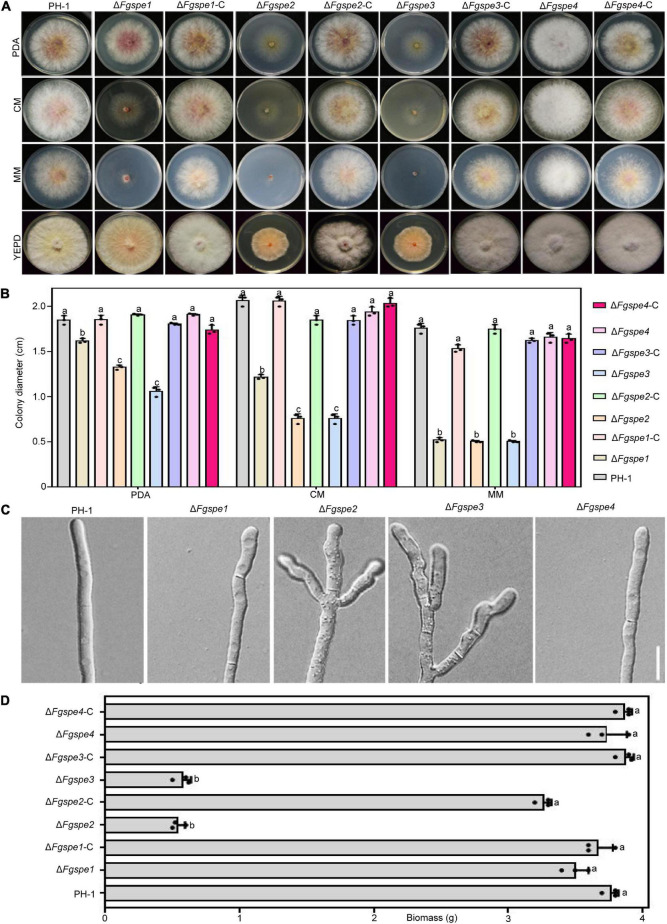
Phenotypes of the Δ*Fgspe* mutants in vegetative growth. **(A)** Colony morphology of wild-type PH-1, the Δ*Fgspe* mutants and complemented strains cultured on PDA, CM, MM, and YEPD medium for 3 days at 25°C. **(B)** Statistical analysis of hyphal growth rate of wild-type PH-1 and the mutants. The diameter was measured after culturing at 25°C for 3 days. Columns listed with the same letter indicates no obviously difference with the least significant difference (LSD) test at *P* = 0.05. **(C)** The patterns of hyphal branching and tip growth for wild-type and Δ*Fgspe* mutants inoculated in PDB liquid medium for 24 h. Bar, 50 μm. **(D)** Statistical analysis of biomass of wild-type, the Δ*Fgspe* mutants, and complemented strains for 2 days in YEPD medium.

Polyamines play an essential role to maintain normal growth and development in a living organism (Igarashi and Kashiwagi, 2019). To further study the function of polyamine biosynthesis gene in *F. graminearum*, we constructed deletion mutants, Δ*Fgspe1*, Δ*Fgspe2*, Δ*Fgspe3*, and Δ*Fgspe4*, using homologous recombination strategy by replacing open reading frame (ORF) of *FgSPE* gene with hygromycin B phosphotransferase (hph) gene cassette. Transformants in each mutant showed consistent phenotypes on the same conditions. To verify that the phenotypic defects in mutants were due to gene deletion, we constructed the complemented vector for each mutant and generated the Δ*Fgspe1*-C, Δ*Fgspe2*-C, Δ*Fgspe3*-C, and Δ*Fgspe4*-C complemented strains. As shown in Figures 2A,B, the loss of Fgspe1 resulted in significantly reduced growth in CM medium and MM medium that were polyamine-free mediums. However, the Δ*Fgspe1* exhibited similar mycelial growth rates compared with the wild-type strain in PDA and YEPD medium that may be included by trace amounts of polyamines. The Δ*Fgspe2* and Δ*Fgspe3* mutants showed obviously hyphal growth defects on PDA, CM, MM, and YEPD medium. Moreover, Δ*Fgspe2* and Δ*Fgspe3* also showed a reduction in aerial hyphae. However, Δ*Fgspe4* showed no obvious phenotypic defects. It might indicate that Fgspe4 encoding spermine synthase was not important for the growth of *F. graminearum* (Figures 2A,B). Subsequently, microscopic observation exhibited that the hyphal morphology of Δ*Fgspe2* and Δ*Fgspe3* mutants were highly branched and misshapen (Figure 2C). The dry weight biomass of Δ*Fgspe2* and Δ*Fgspe3* were significantly reduced compared to wild-type in YEPD medium for 2 days (Figure 2D). As shown in Supplementary Figure 1, Fgspe2 and Fgspe3 are essential for Spd synthesis. Therefore, our result indicates that the endogenous Spd is vital for the hyphal growth of *F. graminearum*.

### Polyamines Restore the Defective Phenotypes of Δ*Fgspe* Mutants

**FIGURE 3:**
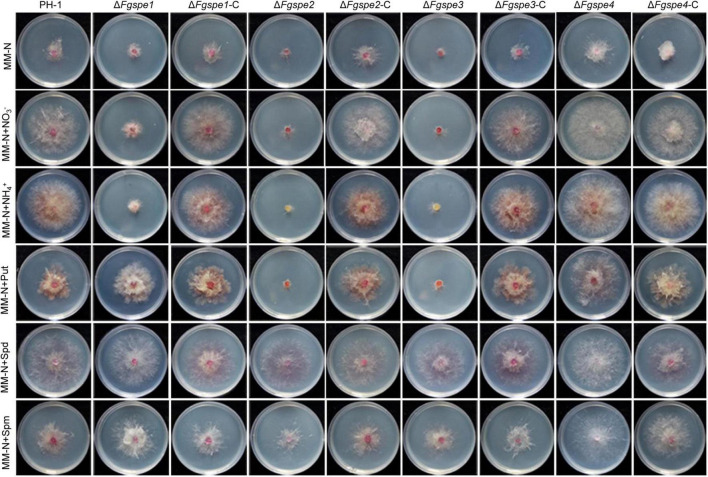
Polyamines restores the growth defection of Δ*Fgspe* mutants. Colony morphology of PH-1, deletion mutants, and complementation mutants in MM-N medium supplemented with different nitrogen sources at 5 mM. Photos were recorded at 25°C after 3 days.

As shown in Figure 3, wild-type PH-1 showed as seriously growth defective in the MM-N medium in which was not included any nitrogen source. To further confirm the function of the polyamine in *F. graminearum* therefore, we firstly testified the growth rate of wild-type PH-1 in MM-N medium by exogenous supplemented polyamine, NO_3_^–^ and NH_4_^+^ served as the control. In Figure 3, the phenotypic defects of wild-type PH-1 in MM-N medium showed better recovery by exogenous supplementation of Spd in comparison with Put and Spm, indicating that Spd exhibits a more important role for the growth of *F. graminearum*. The Δ*Fgspe1*, Δ*Fgspe2*, and Δ*Fgspe3* mutants showed the seriously defective growth in CM, MM, and MM-N mediums that were free of polyamines (Figures 2A, 3). Put, Spd, and Spm are the downstream metabolites of ornithine decarboxylase (Vanrell et al., 2013). To explore the defective phenotypes of Δ*Fgspe* mutants and whether they could be recovered by the addition of exogenous polyamines, the Δ*Fgspe* mutants were grown on MM-N medium supplemented with Put, Spd, and Spm at 5 mM concentration, respectively. NO_3_^–^ and NH_4_^+^ served as the control. The results showed that NO_3_^–^ and NH_4_^+^ were not conducive for growth restoration of Δ*Fgspe1*, Δ*Fgspe2*, and Δ*Fgspe3* mutants. When Put was added to MM-N medium, defects in hyphal growth of Δ*Fgspe1* mutant were restored (Figure 3). When exogenous Spd was added to an MM-N medium, the vegetative growth of the Δ*Fgspe2* and Δ*Fgspe3* could be fully rescued. In general, our data further indicates that the Spd plays a vital role in hyphal growth in this fungus.

### Spermidine Is Essential for Asexual and Sexual Reproduction

**FIGURE 4:**
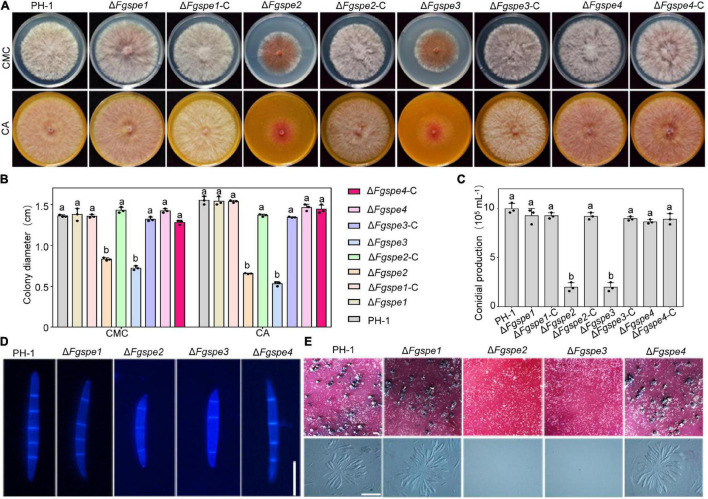
Spermidine is important for asexual and sexual reproduction. **(A)** Colony morphology of PH-1, deletion mutants, and complementation mutants on CMC and CA agar medium. **(B)** The growth rate of mutants after 3 days were analyzed. Columns listed with the same letter are not significantly different at *P* = 0.05. **(C)** The number of conidia produced by each strain in CMC medium for 4 days. **(D)** Conidial morphology of wild-type and Δ*Fgspe* mutants strains. The septa were stained by Calcofluor White and photographed by a fluorescence microscope. Bar, 50 μm. **(E)** Typical perithecia (upper panel), asci, and ascospores (bottom panel) formed by PH-1 and Δ*Fgspe* mutants on CA. Photographs were recorded after incubation for 3 weeks. Bar, 200 μm.

Sexual and asexual reproduction play a vital role in the infection cycle of *F. graminearum*. Subsequently, the conidial and perithecia production of Δ*Fgspe* mutants was observed. Firstly, to rule out the effect of growth defects on asexual and sexual reproduction, the growth of Δ*Fgspe* mutants was determined. As shown in Figures 4A,B, the Δ*Fgspe* mutants and complementary strains showed a consistent phenotype in conidiation (CMC) and sexual reproduction (CA) in this medium in comparison with the PDA medium (Figure 2A). Hence, we further observed the conidial and perithecia production of Δ*Fgspe* mutants in CMC and CA medium. Compared to wild-type, our result indicated that the Δ*Fgspe2* and Δ*Fgspe3* exhibited an obvious reduction in conidiation after incubation for 4 days in CMC medium (Figure 4C). To further observe conidial morphology, the conidia of all mutants were stained by CFW dye. The conidial morphology was recorded with a fluorescence microscope. The conidia size of Δ*Fgspe2* and Δ*Fgspe3* was shorter in comparison with wild-type (Figure 4D). Moreover, the Δ*Fgspe2* and Δ*Fgspe3* showed dramatic reduction in perithecia formation on the CA agar medium in comparison with wild-type (Figure 4E). Thus, our results indicate that endogenous Spd is critical for the asexual and sexual reproduction of *F. graminearum*.

### Spermidine Regulates Environmental Stress Tolerance

**FIGURE 5:**
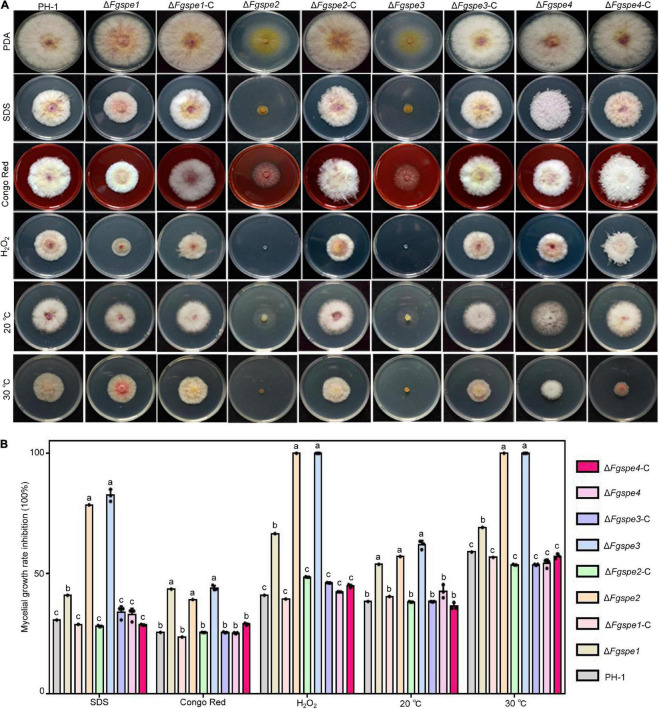
Δ*Fgspe2* and Δ*Fgspe3* mutants have increased sensitivities to environmental stresses. **(A)** Sensitivity assay of deletion mutants to 0.02% SDS, 0.2 g/L Congo Red, 0.02% H_2_O_2_, and different temperatures. **(B)** The mycelial growth inhibition rate of each strain was examined after 3 days on PDA. Columns listed with the same letter are not significantly different at *P* = 0.05.

A previous study conducted by Yamaguchi et al. (2007) has indicated that polyamine is important to the response to various environmental stresses. Therefore, we detected the sensitivities of the Δ*Fgspe* mutants to different environmental stress agents, including sodium dodecyl sulfate (SDS), Congo Red, H_2_O_2_, and temperature. The sensitivity assays indicated that the Δ*Fgspe2* and Δ*Fgspe3* showed significantly increased sensitivity to various stresses compared to the wild-type. Moreover, the Δ*Fgspe2* and Δ*Fgspe3* mutants could not grow at the high temperature and oxidative stress generated by 30°C and H_2_O_2_, respectively. Δ*Fgspe1* shows weakly increased sensitivity to environmental stress in Figure 5A. However, Δ*Fgspe4* showed a similar susceptibility in the wild-type toward the above stress (Figures 5A,B). The complementation strains exhibited the same sensitivities as wild-type under various stress conditions. Aurofusarin of *Fusarium* fungi played an important role in responding to environment adaptation (Xu et al., 2019). Therefore, we observed the aurofusarin production of Δ*Fgspe* mutants and found that Δ*Fgspe2* and Δ*Fgspe3* mutants showed increased yellow pigments on PDA medium (Supplementary Figure 2). In general, our results indicate that Spd shows a vital role in the response to abiotic and biotic stresses.

### Spermidine Is Required for the Virulence of *Fusarium graminearum*

**FIGURE 6:**
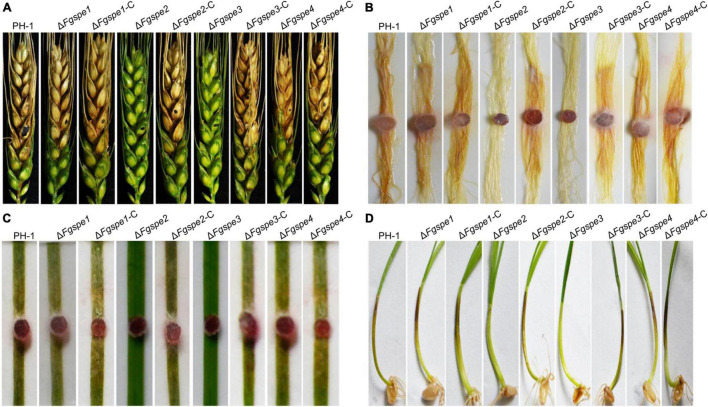
Δ*Fgspe2* and Δ*Fgspe3* mutants have attenuated virulence in plant. **(A)** Pathogenicity of all tested strains on flowering wheat heads. Wheat heads were photographed at 15 days post-inoculation using a conidial suspension. The injection sites were denoted as black dots. **(B)** The Δ*Fgspe2* and Δ*Fgspe3* mutants also showed significantly reduced virulence on corn silks. Corn silks were inoculated with fresh mycelial plug of each strain and were determined after 5 days post-inoculation at 25°C. **(C)** Disease symptoms infected by the tested strains on young wheat leaves. The images were photographed at 6 days. **(D)** Pathogenicity of the wild-type, mutants, and complementation strains to wheat coleoptiles for 7 days.

**FIGURE 7:**
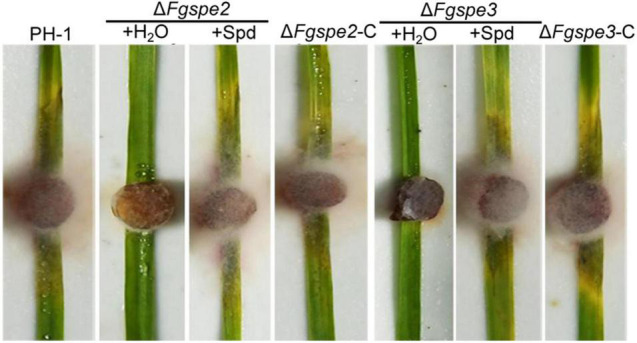
Exogenous addition of spermidine restores virulence of Δ*Fgspe2* and Δ*Fgspe3* mutants in plant. Disease symptom on wheat seedling leaves inoculated with the Δ*Fgspe2* and Δ*Fgspe3* mutants which are treated with or without Spd at the final concentration of 5 mM.

To further investigate the function of polyamine in this pathogenic progress, the virulence of the Δ*Fgspe* mutants was evaluated in flowering wheat heads, the leaves of wheat seedlings, corn silk, and wheat coleoptiles. Notably, the wild-type, Δ*Fgspe1*, Δ*Fgspe4*, and complemented strains could develop on the injected spikelets and quickly spread to the neighboring wheat head, causing discoloration symptoms 2 weeks after inoculation. However, the Δ*Fgspe2* and Δ*Fgspe3* mutants were not capable of penetrating the spikelet (Figure 6A). Additionally, the Δ*Fgspe2* and Δ*Fgspe3* demonstrated attenuated virulence on seedling leaves, corn silk, and wheat coleoptiles (Figures 6B–D). To demonstrate that Spd is the main cause of the reduction in virulence of Δ*Fgspe3*, furthermore, we testified whether exogenous Spd supplement assay could rescue the virulence defects of Δ*Fgspe2* and Δ*Fgspe3*. Our result showed that the addition of exogenous Spd can indeed rescue the virulence defects of the Δ*Fgspe2* and Δ*Fgspe3* mutants *in planta* (Figure 7). These results demonstrate that Spd is important for virulence in *F. graminearum*.

### Spermidine Regulates Deoxynivalenol Biosynthesis

**FIGURE 8:**
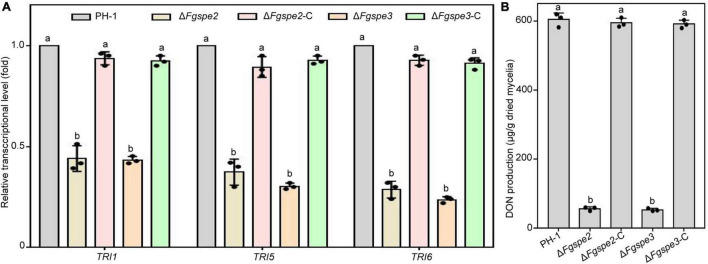
Effects of Δ*Fgspe2* and Δ*Fgspe3* mutants on DON biosynthesis. **(A)** Relative expression levels of *TRI1*, *TRI5*, and *TRI6* in the wild-type PH-1 and Δ*Fgspe3* in TBI medium for 2 days. The *ACTIN* gene was served as an internal control. **(B)** DON contents of the wild-type PH-1 and Δ*Fgspe3* strains after in TBI medium 7 days of incubation. Columns listed with the same letter are not significantly different at *P* = 0.05.

As DON is proposed as a vital virulence factor and assists the fungus spread during *F. graminearum* infection process (Seong et al., 2009), we guessed that the reduced pathogenicity of Δ*Fgspe2* and Δ*Fgspe3* was also partly due to a reduction in DON production. Therefore, the transcriptional levels of *TRI* genes which are essential for DON synthesis in the Δ*Fgspe2* and Δ*Fgspe3* were detected by RT-qPCR assay after incubation for 2 days in liquid TBI medium. These results indicated that the expression levels of *TRI6*, *TRI5*, and *TRI1* genes were significantly down-regulated in the Δ*Fgspe2* and Δ*Fgspe3* compared with wild-type (Figure 8A). Furthermore, the DON synthesis of Δ*Fgspe2* and Δ*Fgspe3* mutants showed a dramatic reduction after incubation for 7 days in a TBI medium (Figure 8B). These results suggest that Δ*Fgspe2* and Δ*Fgspe3* are essential for DON biosynthesis in *F. graminearum*.

### Transcriptional Profiling Analysis of Δ*Fgspe3*

**FIGURE 9:**
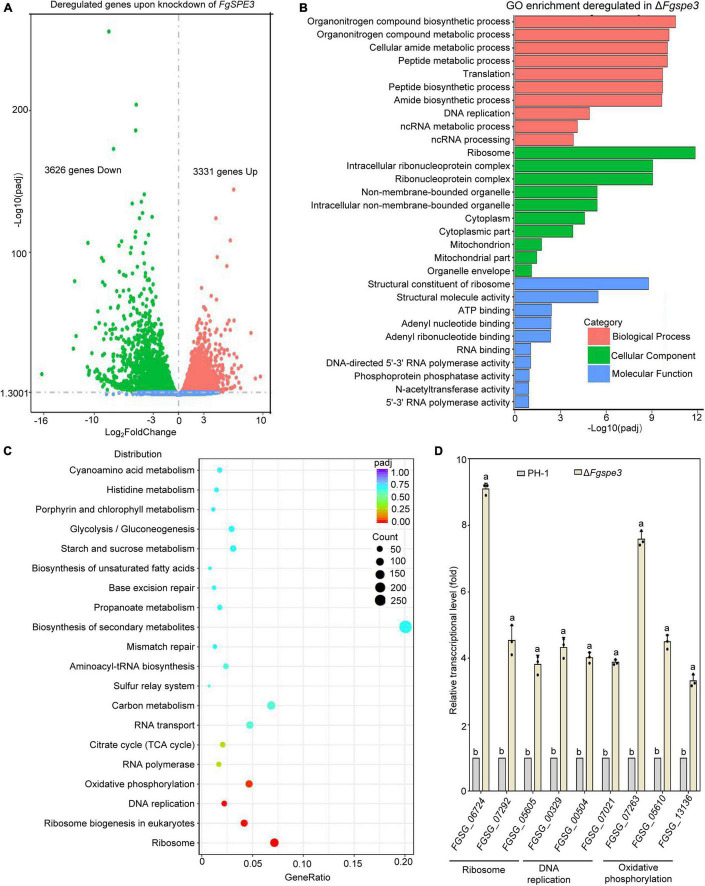
Transcriptomic analyses between Δ*Fgspe3* and the wild-type PH-1. **(A)** Volcano plot analysis of differentially expressed genes (DEGs) of Δ*Fgspe3*. The *x*-axis shows the log2fold-change in RNA expression between PH-1 and Δ*Fgspe3*, while the *y*-axis indicates the –log10 of the adjusted *P*-value for each RNA, The *P*-values were adjusted using Benjamini–Hochberg multiple testing. The red dots and green dots represent upregulated and downregulated DEGs. **(B)** Gene Ontology (GO) term enrichment analysis of DEGs of Δ*Fgspe3*. **(C)** Kyoto Encyclopedia of Genes and Genomes (KEGG) pathway enrichment analysis of DEGs of Δ*Fgspe3*. **(D)** Expression levels of selected genes were detected by RT-qPCR to PH-1 and Δ*Fgspe3* mutant strains in YEPD medium for 48 h. Results are shown relative to PH-1 and are normalized to the housekeeping gene *ACTIN*. Columns listed with the same letter are not significantly different at *P* = 0.05.

To further explore the function of Spd, we performed RNA-seq to wild-type and Δ*Fgspe3* strain in YEPD medium. For each strain, we plotted three biological replicates. For most genes, the replicates produced similar FPKM values, and all points fall near a line with a slope of 1 (Supplementary Figure 3). Transcriptome analysis revealed that 6,957 genes were differentially expressed in the Δ*Fgspe3* compared with the wild-type, including 3,331 up-regulated and 3,626 down-regulated genes (Figure 9A). Gene Ontology (GO) and Kyoto Encyclopedia of Genes and Genomes (KEGG) pathway enrichment analyses were performed to identify the function of the differentially expressed genes (DEGs). GO enrichment analysis identified that genes that are significantly expressed in the Δ*Fgspe3* mutant were mainly involved in biological processes such as organonitrogen compound biosynthetic, peptide metabolic and translation; cellular components such as ribosome, intracellular ribonucleoprotein complex, cytoplasm, and mitochondrion; and molecular functions such as structural constituents of ribosomes, and RNA and ATP binding (Figure 9B). Furthermore, KEGG enrichment analysis showed a list of the top 20 regulated pathways in Δ*Fgspe3*, including ribosome, DNA replication, oxidative phosphorylation, RNA polymerase, and the citrate cycle (TCA cycle) (Figure 9C). Then, we tested transcriptional expression patterns of nine representative DEGs by qRT-PCR, and the trends in gene expression profiles from qRT-PCR were positively correlated with the transcriptomic data, indicating that expression levels based on RNA-seq data were believable (Figure 9D). Taken together, these results indicate that Fgspe3 might modulate the essential cellular process involved in ribosomes, DNA replication, and carbon metabolism in *F. graminearum*.

## Discussion

As an important class of metabolites, the polyamines are found distributed in almost all organisms and participate in multiple biological processes, including gene expression regulation, translation, stress resistance, autophagy, and pathogenesis (Miller-Fleming et al., 2015; El-Tarabily et al., 2020, 2021; Mathew et al., 2020). In the current study, our data generated from knocking out Fgspe3 agree with previous publications. Majumdar et al. (2018) have reported that Spd is essential for growth, development, the production of aflatoxins, and pathogenesis in *A. flavus*. Consequently, difluoromethylarginine (DFMA) and L-α-difluoromethylornithine (DFMO) inhibitors of ODC and ADC, respectively, can significantly decrease the fungal growth and virulence (Gamarnik et al., 1994). In *F. graminearum*, DFMO could inhibit growth and DON production *in vitro*. Additionally, some polyamine transport inhibitors were also shown to be effective against *F. graminearum* activity (Crespo-Sempere et al., 2015). In this study, the genes of polyamines biosynthetic pathway have been identified in *F. graminearum*. The data presented in this study are in line with earlier reports on the role of polyamines in *A. flavus* and demonstrate that polyamines are important for growth, development, and stress resistance in *F. graminearum*. Importantly, the metabolic pathways of polyamines are different in plants and fungi. Thus, targeted and precision-regulated polyamines biosynthetic pathway might represent a novel therapeutic strategy to control plant and animal diseases caused by some phytopathogenic fungi (Valdés-Santiago et al., 2012; Amano et al., 2015).

The current study also shows that supplementation of external Spd can extend the lifespan of a range of species, including yeast, flies, and mice. It has been reported that Spd may be an anti-aging vitamin for humans, and deterioration from aging may be associated with a reduction in endogenous Spd (Madeo et al., 2018, 2019). In many cases, polyamines are most closely associated with stress tolerance (El-Tarabily et al., 2020, 2021; Mathew et al., 2020). The accumulation of endogenous polyamines is crucial for organisms in response to external stress (Shen et al., 2000). Because of the polycationic nature, polyamines may also stabilize the membranes by interacting with membrane phospholipids under stress conditions (Roberts et al., 1986). Polyamines can also enhance stress tolerance that may be due to regulating nutrient balance and production of reactive oxygen species (ROS) through sophisticated signaling systems (Gong et al., 2014). In this study, the Δ*Fgspe3* mutant was hypersensitive to H_2_O_2_ and high temperature. It might indicate that the strong antioxidant capability of polyamines results in the scavenging of ROS on oxidative stress conditions (Minocha et al., 2014). In this study, the Δ*Fgspe3* mutant showed significantly increased sensitivity to the tested environmental stress compared with wild-type. These results indicate that the polyamine of *F. graminearum*, especially Spd, plays a crucial role in responding to environmental stresses.

In transcription analysis, genes with log_2_ Fold change > 1 or < −1 and adjusted *P-*value < 0.05 as the cutoff criterion were selected for subsequent analysis. Our results *FgSPE3* participated in many important biological processes such as peptide metabolic, organonitrogen compound biosynthetic, and translation; cellular components such as intracellular ribonucleoprotein complex, intracellular non-membrane-bounded organelle, cytoplasm, and mitochondrion; and molecular function such as structural constituent of ribosome, RNA and ATP binding. In agreement with a previous study (Igarashi and Kashiwagi, 2000), Polyamines are positively charged molecules and thus can interact with intracellular anionic macromolecules, such as DNA, RNA, chromatin, proteins, eukaryotic translation initiation factor 5A (eIF5A), and phospholipids. Thereby they might potentially affect the outcome of several downstream cellular processes, including subsequent changes in the chromatin structure that can affect gene transcription and eIF5A hypusine modification can regulate the protein translation (Watanabe et al., 1991; Rocha and Wilson, 2019). A growing number of studies have indicated that polyamines play a critical role in the methylation and acetylation of histones, RNA, and DNA, and chromatin remodeling (Phadwal et al., 2018; Tamari et al., 2018). Together, these results further testify to our finding that Spd plays vital roles in hyphal growth and development in *F. graminearum*. Further characterization of the functions of those genes of polyamine biosynthetic pathway will help to better understand the roles of polyamines in this fungus.

In the rice blast fungus *M. oryzae*, spermine metabolism was identified to be associated with infection-related morphogenesis *via* redox buffering of the ER underpinning appressorial adhesion and rice cell invasion (Rocha et al., 2020). Another role of polyamines was also characterized in FHB caused by *F. graminearum* (Gardiner et al., 2010). Interestingly, the production of DON is significantly induced by several plant metabolites including Put, ornithine, and arginine which are the main natural precursors of polyamines synthesis (Gardiner et al., 2009a). However, the Fgspe4, encoding spermine synthase, showed no obvious defective phenotypes including growth, development, and pathogenesis. These results show that the functions of polyamine vary dramatically among fungal species. We found, in this study, that Δ*Fgspe2* and Δ*Fgspe3* mutants were non-pathogenic. The DON production level of Δ*Fgspe2* and Δ*Fgspe3* mutants were significantly reduced in comparison with wild-type. DON is indispensable for the pathogen to extend from infected head to healthy head (Gardiner et al., 2009b). It may be indicated the endogenous spermidine is critical for infection of *F. graminearum*. Additionally, the slower growth of the Δ*Fgspe2* and Δ*Fgspe3* mutants may be partially responsible for the reduced virulence in plants. Whether the reduction in pathogenicity in Δ*Fgspe2* and Δ*Fgspe3* mutants during infection is due to the decrease in Spd or possible alteration in relative DON content or both, will require further investigation. Overall the results suggest polyamine biosynthesis process is an attractive target to control FHB and reduce DON contamination.

## Data Availability Statement

The datasets presented in this study can be found in online repositories. The names of the repository/repositories and accession number(s) can be found below: https://www.ncbi.nlm.nih.gov/, PRJNA759842.

## Author Contributions

GT, ZM, and WL: conceptualization, data analysis, and writing the manuscript. GT, HX, and JL: experiments. All authors have read and agreed to the published version of the manuscript.

## Conflict of Interest

The authors declare that the research was conducted in the absence of any commercial or financial relationships that could be construed as a potential conflict of interest. The handling editor declared a shared affiliation all of the authors at the time of review.

## Publisher’s Note

All claims expressed in this article are solely those of the authors and do not necessarily represent those of their affiliated organizations, or those of the publisher, the editors and the reviewers. Any product that may be evaluated in this article, or claim that may be made by its manufacturer, is not guaranteed or endorsed by the publisher.

## Footnotes

1
https://www.ncbi.nlm.nih.gov/sra/

## Supplementary Material

Supplementary Figure 1 Polyamines biosynthetic pathway.

Supplementary Figure 2 Comparisons of pigment biosynthesis of wild-type and Δ*Fgspe* mutants generated in this study. Photos were recorded after each strain was grown for 7 days on a PDA medium.

Supplementary Figure 3 Global transcriptional analysis. **(A)** Biological replicates are highly correlated. Boxplot represents FPKM distributions of different samples. The *y*-axis is log2 (FPKM + 1). **(B)** Gene expression levels are displayed at different FPKM levels. The *x*-axis indicates log2 (FPKM + 1), and the y axis shows the density of the gene.
