# Supplementary figures and images for: Spermidine Is Critical for Growth, Development, Environmental Adaptation, and Virulence in Fusarium graminearum
Source: Front Microbiol. 2021 Nov 19;12:765398. doi: 10.3389/fmicb.2021.765398 (PMC8640359; doi:10.3389/fmicb.2021.765398)

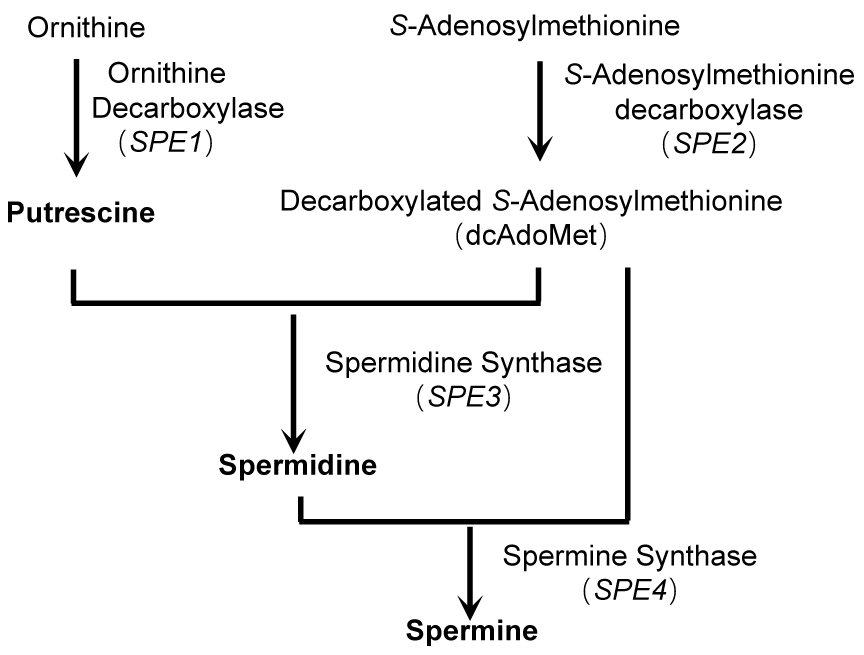

Supplement: Supplementary Figure 1 — Polyamines biosynthetic pathway. [file Image_1.TIF]

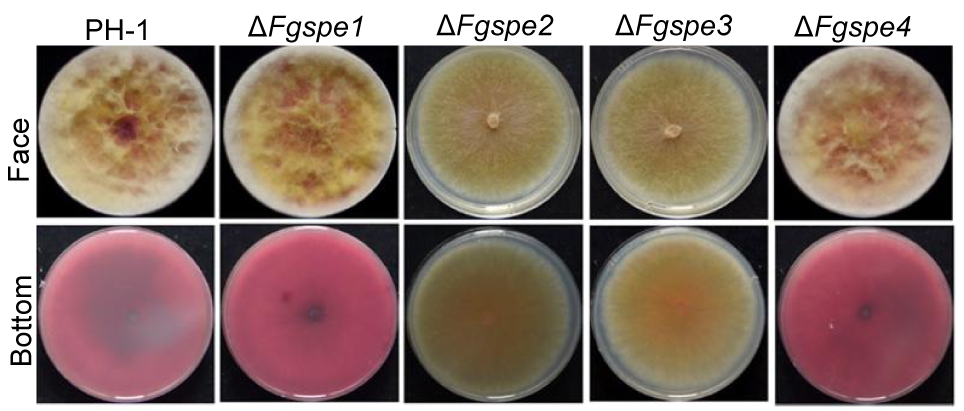

Supplement: Supplementary Figure 2 — Comparisons of pigment biosynthesis of wild-type and ΔFgspe mutants generated in this study. Photos were recorded after each strain was grown for 7 days on a PDA medium. [file Image_2.TIF]

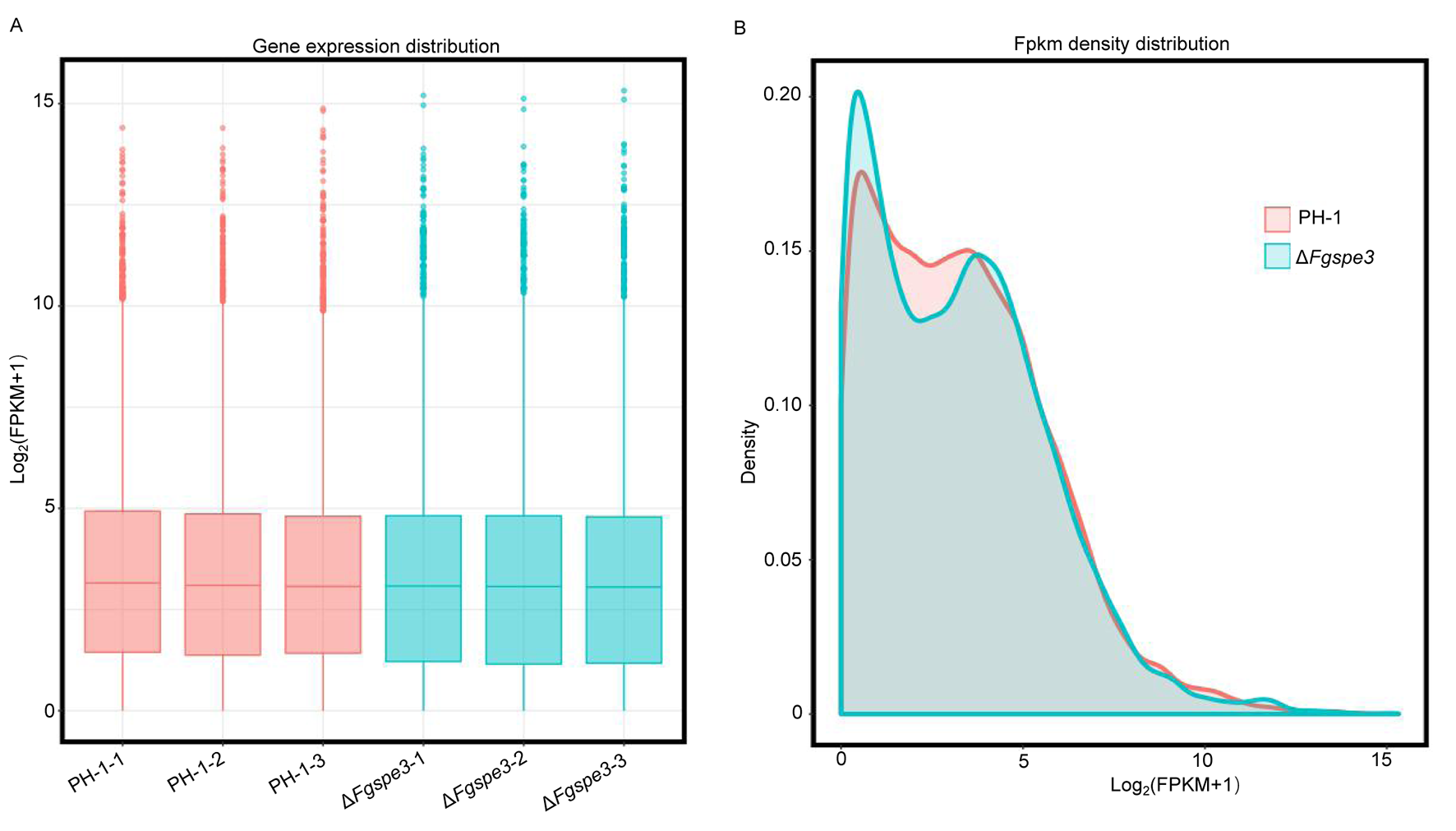

Supplement: Supplementary Figure 3 — Global transcriptional analysis. (A) Biological replicates are highly correlated. Boxplot represents FPKM distributions of different samples. The y-axis is log2 (FPKM + 1). (B) Gene expression levels are displayed at different FPKM levels. The x-axis indicates log2 (FPKM + 1), and the y axis shows the density of the gene. [file Image_3.TIF]
