# Supplementary material for: Spermidine Is Critical for Growth, Development, Environmental Adaptation, and Virulence in Fusarium graminearum
Source: Front Microbiol. 2021 Nov 19;12:765398. doi: 10.3389/fmicb.2021.765398 (PMC8640359; doi:10.3389/fmicb.2021.765398)
Supplement: Supplementary file 4 [file Table_1.doc]

**Table 1 A list of primers used in this study**

| Code | Name | Sequence (5'to 3') | Products |
| --- | --- | --- | --- |
| 1 | Fgspe1-P1 | GGATTGGGTGGTGTTTACGA | Upstream fragment of Fgspe1 |
| 2 | Fgspe1-P2 | CAAAATAGGCATTGATGTGTTGACCTCCTGTGGTAGGAGTGTAACCGAAA |
| 3 | Fgspe1-P3 | CTCGTCCGAGGGCAAAGGAATAGAGTAGACCAGCAGCCCACCGCCAAGAT | Downstream fragment of Fgspe1 |
| 4 | Fgspe1-P4 | TGACGACAAGGCTATGGAAT |
| 5 | Fgspe1-ID-F | AGGCAACATCTTCACTACGGC | Identification Fgspe1deletion transformants |
| 6 | Fgspe2-ID-R | CCTCAAAGTAAAGCCAGTCG |
| 7 | Fgspe2-P1 | GTCGTCACCCGAAAGAGGCT | Upstream fragment of Fgspe2 |
| 8 | Fgspe2-P2 | CAAAATAGGCATTGATGTGTTGACCTCCGGAGATTTCTTTGCCCAGAT |
| 9 | Fgspe2-P3 | CTCGTCCGAGGGCAAAGGAATAGAGTAGCGTCTACCCAACATCCAAGTATCCC | Downstream fragment of Fgspe2 |
| 10 | Fgspe2-P4 | GGAGTATTGTTGTTGCGGTAGGTT |
| 11 | Fgspe2-ID-F | TCCGCCAGCATCTGAACAAG | Identification Fgspe2 deletion transformants |
| 12 | Fgspe2-ID-R | CGAGCAATTTATTCCTCCTCTG |
| 13 | Fgspe3-P1 | CAGCACACTTCCTCCTCTTT | Upstream fragment of Fgspe3 |
| 14 | Fgspe3-P2 | CAAAATAGGCATTGATGTGTTGACCTCCAGTGACGATGTAGTTGTGGT |
| 15 | Fgspe3-P3 | CTCGTCCGAGGGCAAAGGAATAGAGTAG GAACGGCAGAATCAGGGAAC | Downstream fragment of Fgspe3 |
| 16 | Fgspe3-P4 | AGGTTGAGGCGGACGAAAAG |
| 17 | Fgspe3-ID-F | GCCTGATATCGTCTGGGGCA | Identification Fgspe3 deletion transformants |
| 18 | Fgspe3-ID-R | GCTGACATATACGCTTGAT |
| 19 | Fgspe4-P1 | AGTGCTATGCGTAGTCCACG | Upstream fragment of Fgspe4 |
| 20 | Fgspe4-P2 | CAAAATAGGCATTGATGTGTTGACCTCC TTTGTTTCTGAGTGGTGGCT |
| 21 | Fgspe4-P3 | CTCGTCCGAGGGCAAAGGAATAGAGTAG ATCGTCTTGGCTCCTATTAC | Downstream fragment of Fgspe4 |
| 22 | Fgspe4-P4 | CCACGAGCACGCAGCAAAAT |
| 23 | Fgspe4-ID-F | TGTCAGTGGCTGATTTGAAC | Identification Fgspe4 deletion transformants |
| 24 | Fgspe4-ID-R | GGCTTGCCACTTCATATGG |
| 25 | Np-spe1-GFP-F: | GACCTCGAGGGGGGGCCCGGATTGGGTGGTGTTTACGA | A pair of PCR primers to amplify *SPE1* fragments used for construction of the Fgspe1-GFP, vector under its own promoter |
| 26 | Np-spe1-GFP-R: | CTCCTCGCCCTTGCTCACCATTAGTCCGAGAAGGGCCTTGG |
| 27 | Np-spe2-GFP-F: | GACCTCGAGGGGGGGCCCGTCGTCACCCGAAAGAGGCT | A pair of PCR primers to amplify *SPE2* fragments used for construction of the Fgspe2-GFP, vector under its own promoter |
| 28 | Np-spe2-GFP-R: | CTCCTCGCCCTTGCTCACCATCTTGAAAGTAGGGCTGATAG |
| 29 | Np-spe3-GFP-F: | GACCTCGAGGGGGGGCCCCTGCTTCAACCTGGTGCAAG | A pair of PCR primers to amplify *SPE3* fragments used for construction of the Fgspe3-GFP, vector under its own promoter |
| 30 | Np-spe3-GFP-R: | CTCCTCGCCCTTGCTCACCATCTCAAGAGCCTTGGCAGCGA |
| 31 | Np-spe4-GFP-F: | GACCTCGAGGGGGGGCCCAGCAAGACGACATACACGAC | A pair of PCR primers to amplify *SPE4* fragments used for construction of the Fgspe4-GFP, vector under its own promoter |
| 32 | Np-spe4-GFP-R: | CTCCTCGCCCTTGCTCACCATCCACTGTTCCCAGACCTTAC |
| 33 | RT-Actin-F | ATCCACGTCACCACTTTCAA | qRT-PCR primers of *ACTIN* |
| 34 | RT-Actin-R | TGCTTGGAGATCCACATTTG |
| 35 | RT-TRI1-F | ACCACCCGCCAAACCCTC | qRT-PCR primers of *TRI1* |
| 36 | RT-TRI1-R | TTCAACAATGGGAGTGATTAG |
| 37 | RT-TRI5-F | ATGGAAAACTTTCCCACCGAG | qRT-PCR primers of *TRI5* |
| 38 | RT-TRI5-R | AGGGTCTACCTTGAGCATCT |
| 39 | RT-TRI6-F | ATGATTTACATGGAGGCCGA | qRT-PCR primers of *TRI6* |
| 40 | RT-TRI6-R | AGATCTATTTCGAATGTTGG |
